# Preoperative Re-evaluation of Planned Orthognathic Surgical Procedures

**DOI:** 10.7759/cureus.86949

**Published:** 2025-06-29

**Authors:** Mami Mutoh, Ko Nakanishi, Saori Tsuchiya, Machiko Kasai, Kazuhiro Matsushita, Takaaki Yamamoto, Yoshiaki Sato

**Affiliations:** 1 Department of Orthodontics, Faculty of Dental Medicine and Graduate School of Dental Medicine, Hokkaido University, Sapporo, JPN; 2 Department of Biomaterials and Bioengineering, Faculty of Dental Medicine, Hokkaido University, Sapporo, JPN; 3 Stomatognathic Function, Center for Advanced Oral Medicine, Hokkaido University Hospital, Sapporo, JPN

**Keywords:** diagnosis, jaw deformity, orthodontic surgery, retrospective study, treatment plan

## Abstract

Objective: In surgical orthodontic treatment, a treatment plan is initially formulated. This plan is reviewed prior to surgery, and the surgical technique is often changed. This study aimed to provide insights into the surgical orthodontic treatment modification process.

Methods: We included 501 patients who visited the Dental Treatment Center of Hokkaido University Hospital from April 2005 to March 2020 and underwent orthognathic surgery by March 2024. The survey items included whether the surgical technique had been changed, the details of any surgical changes, the discussion during the initial consultation, and the reasons for changing the surgical technique. The results were divided into three 5-year periods.

Results: The surgical technique was changed in 138 cases (27.5%). The most common reasons for the change were jaw width, the amount of jaw movement, two occlusal planes, the amount of rotation or cant, and patient condition. Changes may also be attributed to the expanded scope of the surgical support at the time of initial planning and the introduction of insurance for anchor screws. The addition or omission of LeFort I was the most frequent surgical modification.

Conclusions: In treating jaw deformities, the initial treatment should not be strictly adhered to without a preoperative review. Surgical planning should always be considered to reduce the patient's burden and optimize the stability of the occlusion.

## Introduction

Surgical orthodontic treatment is often planned during the initial examination. The surgical plan is re-evaluated, and the final plan is determined just before surgery [1]. In the preoperative review process, the initial surgical plan may be changed. Changes may be required when observations differ between the orthodontic treatment plan and the preoperative review, such as the position of the incisor teeth [2] and medical conditions (systemic disease or pregnancy) [3-5]. Changes may also be indicated for obtaining a more stable occlusion [6]. This does not mean the first operation plan is less optimal.

At Hokkaido University Hospital, three departments (Oral Surgery, Orthodontics, and Prosthodontics) collaboratively plan treatment for patients with jaw deformities. The Department of Orthodontics is responsible for establishing and managing the overall treatment plan, from preoperative orthodontics to the retention phase. The Department of Oral Surgery evaluates the appropriateness and safety of the surgical approach then selects the most suitable surgical plan. The Department of Prosthodontics evaluates occlusion on full mouth support and masticatory function, contributing to the design of the final prosthetic rehabilitation, including the restoration of missing teeth, where necessary.

After the orthodontist interviews the patient and obtains information regarding the chief complaint and medical history, physical examination, facial measurement evaluation, cephalometric analysis, panoramic radiography, computed tomography (CT), and working dentition and jaw movement assessments are performed. After the intra-orthodontic conferences, an inter-department (Orthodontics, Oral Surgery, and Prosthodontics) conference is held to discuss the proposed treatment plan, including the surgical plan. In addition, in all cases, the surgical modalities are reviewed again at a joint conference several months prior to surgery and immediately before surgery. These efforts have ensured the quality of surgical orthodontic treatment even in our hospital with many dentists. Even with this meticulous planning, the surgical plan is often changed due to various factors during the preoperative orthodontic period.

With the expanding age range of patients undergoing surgical orthodontic treatment and the diversity of surgical methods and preoperative orthodontic methods [7-11], preoperative re-evaluation has become increasingly important. Many methods of orthognathic surgery and their resulting stability have been reported [12,13]. To date, changes in surgical plans have not been widely published. Re-evaluation is important because it leads to more optimized orthognathic surgical techniques and greater occlusal stability. Considering the surgical risks and treatment duration, it stands to reason that the more predictable the surgical procedure at the initial consultation, the better the patient outcome. In this study, we evaluated the factors corresponding to surgical plan changes at the preoperative review among patients requiring surgical orthodontic treatment.

## Materials and methods

**Figure 1:**
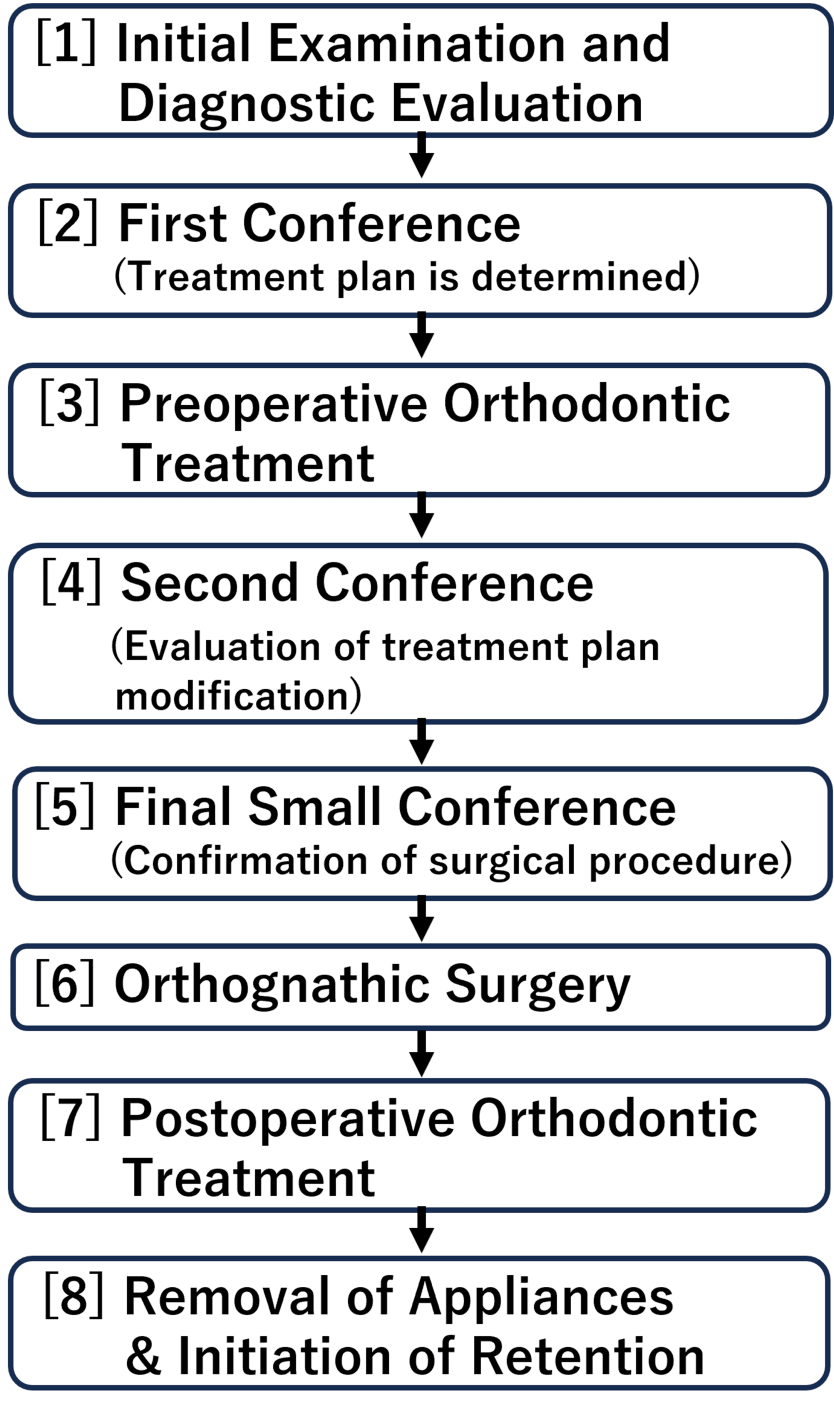
Flowchart of the standard treatment protocol for jaw deformities at Hokkaido University Hospital. The treatment plan is determined at the first multidisciplinary conference, revised if necessary at the second conference, and finalized in the final small conference. Major changes in the treatment plan are rarely made after the second conference.

From April 2005 to March 2020, 665 patients (218 males and 447 females; mean age: 25.5 years) with jaw deformities were presented during the orthognathic surgical conferences at Hokkaido University Hospital (Figure 1). Among the 665 patients, 501 patients who had no congenital deformities or syndromes and underwent surgery by March 2024 were included as study participants.

The participants were surveyed regarding any differences between the initial surgical plan and the completed surgical operation, and the percentage of patients whose surgical procedure was changed was calculated. To determine the causes for patients whose surgery procedure was changed, the inter-canine width, the width between the first permanent molars, tooth axis deviations, and the extent of anterior teeth retroclination were measured using initial and immediate preoperative models, cephalometric radiograms, CT, and medical record. The causes were classified into “width,” “movement,” “two occlusal planes,” “rotation,” “health condition,” “cant,” and “others.” “Width” is the width of the dental arches of the maxilla and mandible, which was the reason for changing the surgical procedure. “Movement” is the amount of maxilla and mandibular movement by orthodontic surgery. “Two occlusal planes” is the difference in the upper occlusal plane between the anterior and posterior teeth. “Rotation” is the amount of mandibular rotation by orthodontic surgery in the sagittal plane. “Health condition” is the patient's health conditions, such as systemic disease and pregnancy. “Cant” is the amount of angulation of the occlusal plane on the frontal plane. The percentage for each cause was calculated. In addition, for cases caused by the width of the maxilla and mandible, we conducted a more detailed investigation into the cause.

The study was divided into three 5-year periods to examine the changes over time. Period 1 (from April 2005 to March 2010): Of the 184 patients with jaw deformities (70 males and 114 females; age at first visit: 22.7 years), 138 underwent orthognathic surgery. Period 2 (from April 2010 to March 2015): Orthognathic surgery was performed on 161 of 224 patients with jaw deformities (74 males and 150 females; age at initial examination: 26.7 years). Period 3 (from April 2015 to March 2020): In total, 202 orthognathic surgeries were performed among 257 patients with jaw deformities (74 males and 183 females; age at initial examination: 27.1 years).

In cases where the surgical procedure was changed, the evaluation of whether the surgery became complicated or simplified was conducted. Complexification refers to cases where surgery on either the maxilla or the mandible alone was changed to surgery on both the maxilla and mandible, maxilla division surgery was added, or the number of maxilla divisions was increased. On the contrary, simplification refers to cases where surgery on both the maxilla and mandible was changed to surgery on either the maxilla or the mandible alone, the maxilla no longer needs to be divided, or the number of maxilla divisions was reduced. The percentage of surgical procedures that had been complicated or simplified was calculated.

This study was approved by the Ethics Committee of Hokkaido University Hospital (Reference number: 016-0170). The option to refuse to participate in this research was given to all participants. The conduct of the research, including the purpose and details, were published and available to all participants.

## Results

Ratio of surgical method change

**Figure 2:**
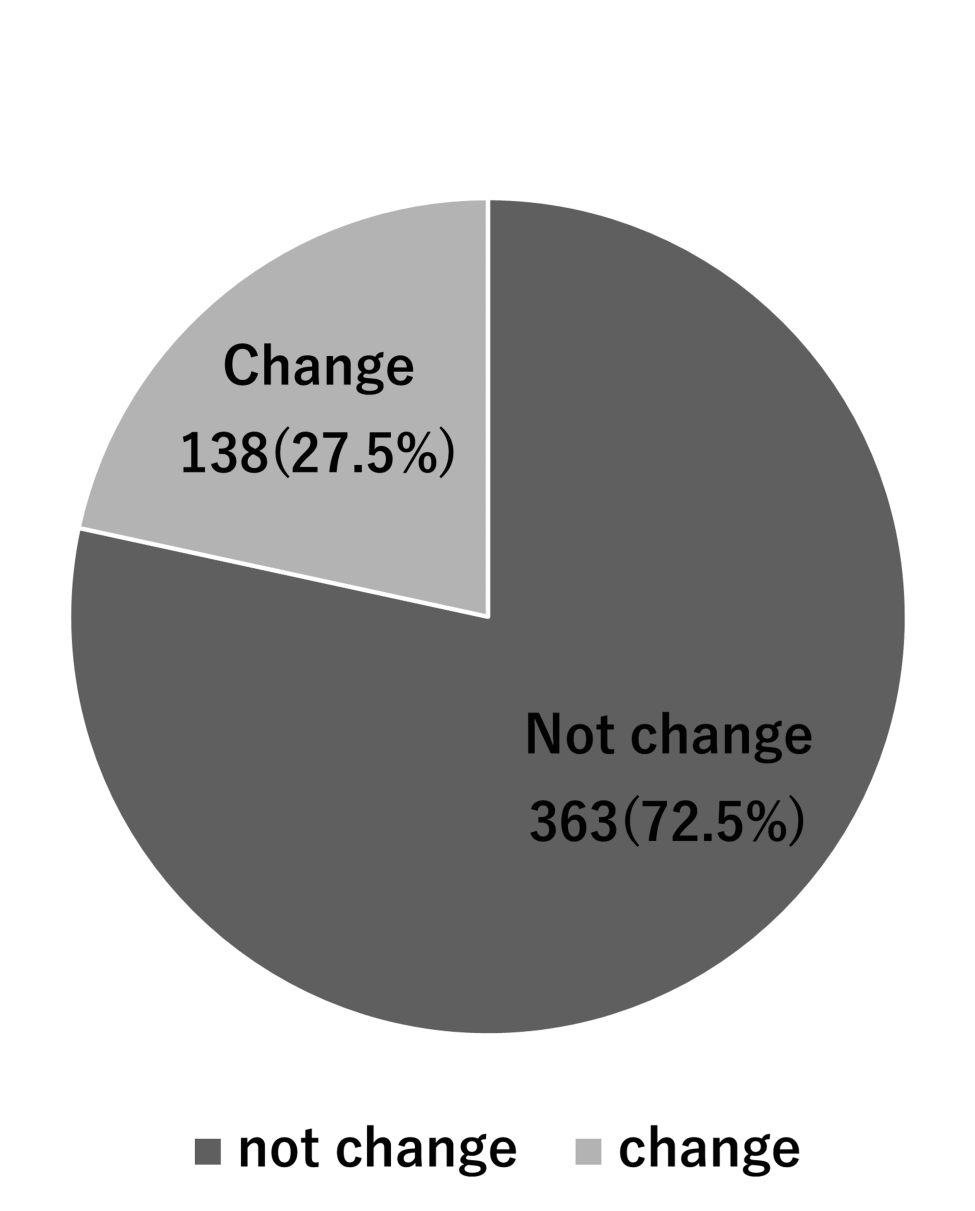
Percentage of the cases where the surgical plan was changed and not changed.

**Figure 3:**
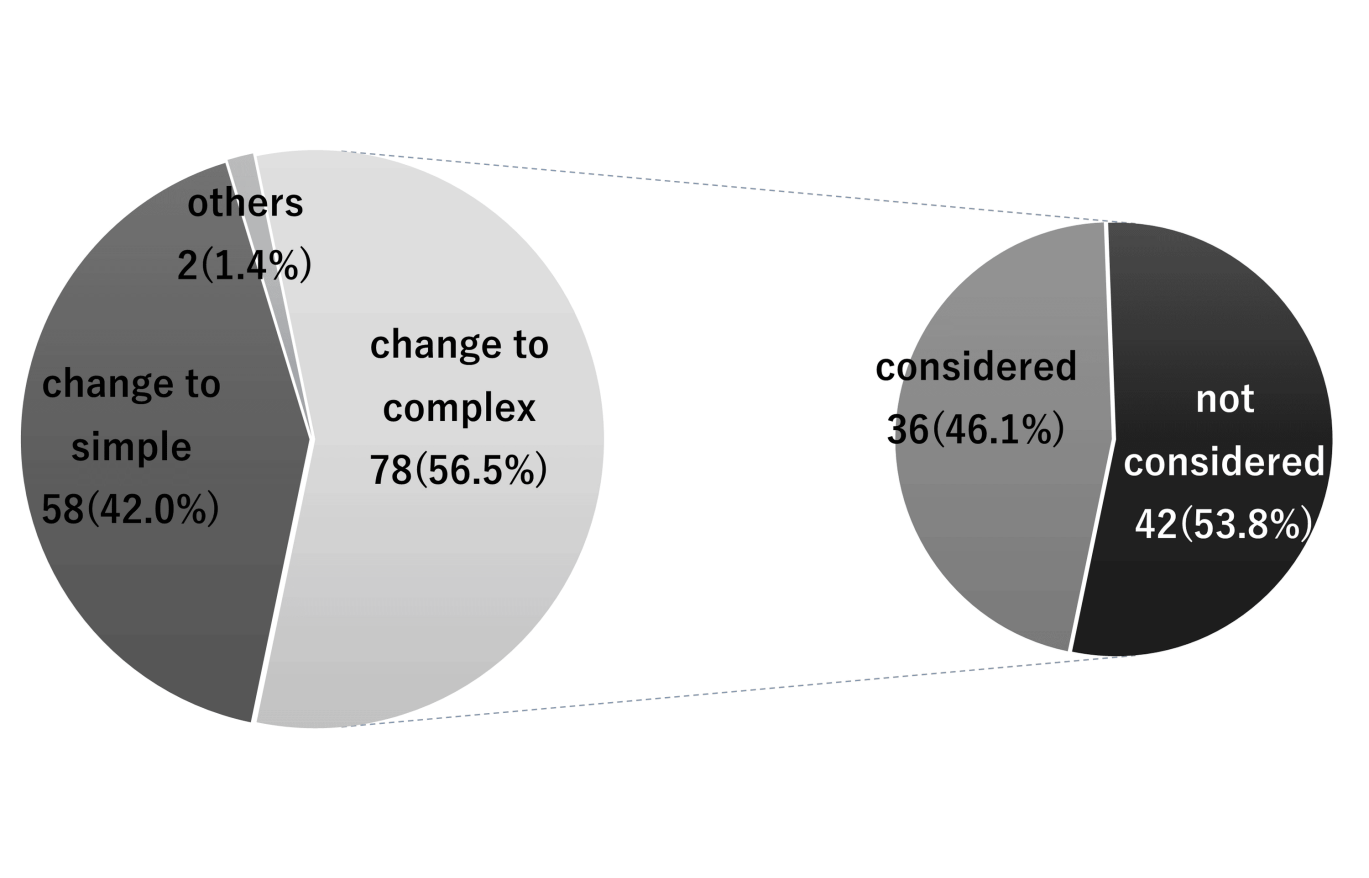
(Left) Percentage of cases resulting in a simple or complex procedure among those that changed. Others refer to cases where there is a change in technique but no change in complexity, such as SSRO changed to LFI. (Right) Whether or not prior consideration was given to the changed procedure among the cases with complex procedures. SSRO: sagittal split ramus osteotomy, LFI: LeFort I.

The surgical plan was changed following the preoperational examination in 27.5% of our participants (138 out of 501 cases) (Figure 2). The surgical method was more complicated than the initial surgical plan in 56.5% (78 of 138 cases). Notably, a more complicated case was defined in this study, in which the surgical method was changed from sagittal split ramus osteotomy (SSRO) only to two jaw surgeries or from a LeFort I monoblock to a multi-split technique. Furthermore, in 36 of 78 patients (46.2%), the surgical method was discussed at the initial examination, including the possible methodological changes (Figure 3).

**Figure 4:**
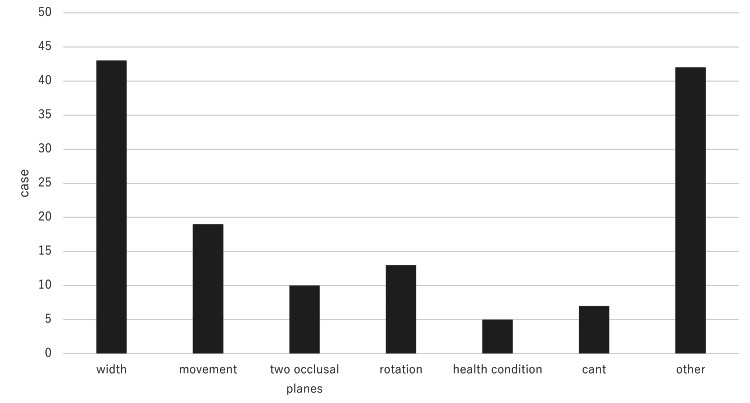
Factors associated with surgical plan changes.

Factors associated with surgical method changes are as follows: arch width (43 cases, 31.1%), jaw movement amount required during surgery (19 cases, 13.8%), mandibular rotation in the sagittal plane required during surgery (13 cases, 9.4%), occlusal plane difference between the anterior teeth and molars (10 cases, 7.2%), occlusal cant (6 cases, 4.3%), and exiting medical/health conditions (7 cases, 5.1%). Other factors related to teeth movement include root resorption and ankylosis (multiple answers allowed) (Figure 4).

Changes by period

**Figure 5:**
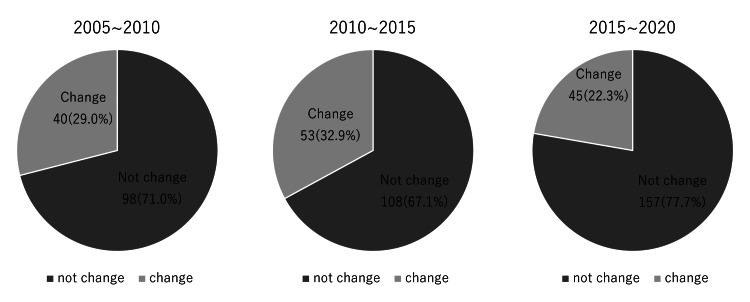
Percentage of patients with change and no change in the surgical plan during the three 5-year periods: (1) 2005-2010, (2) 2010-2015, and (3) 2015-2020. The number of cases per five-year period increased to 138, 161, and 202, while the percentage of patients with changes to the surgical plan increased in the second period and decreased in the third period.

The study was divided into three 5-year periods: (1) 2005-2010, (2) 2010-2015, and (3) 2015-2020. Changes in surgical techniques occurred in 40 patients (29.0%) in Period 1, 53 patients (32.9%) in Period 2, and 45 patients (22.3%) in Period 3. There was no notable change in the ratio of complex to simple techniques (Figure 5). The reasons differed from period to period: 52.3% of cases in Period 1 were due to width diameter changes; 34.6% were due to the amount of movement required and 24.1% were due to width diameter changes in Period 2; and 26.9% were due to the width diameter and the amount of movement in Period 3.

The percentage of changes in surgical procedures decreased in Period 3 compared to Periods 1 and 2. The reasons for change tended to differ according to complexity or simplicity. In Periods 2 and 3, complex changes were related to the amount of jaw movement increased, while the reasons related to the width diameter decreased. Simplification was related to a decrease in width diameter and the increased amount of rotation in Period 3 compared to Periods 1 and 2 (Table 1).

**Table 1:** Reason for complicated and simplified cases.

		Period 1	Period 2	Period 3
Complicated	Width	12 (52.3%)	7 (24.1%)	7 (26.9%)
	Amount	1 (4.3%)	10 (34.6%)	7 (26.9%)
	Two occlusal plane	2 (8.6%)	0	1 (3.8%)
	Rotation	1 (4.3%)	2 (6.9%)	0
	Cant	1 (4.3%)	1 (3.4%)	4 (15.4%)
	Patient's condition	0	0	1 (3.8%)
	Other	6 (26.2%)	9 (31.0%)	9 (34.6%)
Simple	Width	5 (29.3 %)	10 (41.6%)	2 (10.5%)
	Amount	0	1 (4.2%)	0
	Two occlusal plane	6 (35.5%)	1 (4.2%)	0
	Rotation	2 (11.1%)	1 (4.2%)	7 (36.5%)
	Cant	0	0	1 (5.3%)
	Patient's condition	2 (11.8%)	3 (12.5%)	1 (5.3%)
	Other	2 (11.8%)	8 (33.3%)	8 (42.1%)

Factors associated with changes in surgical methods

Width (Transverse Arch Coordination)

In the 43 cases requiring a change in surgical methods due to width problems, the surgical changes involved splitting the maxilla (complicated case) due to a narrower maxilla or wider mandible than expected or, conversely, omitting the need for splitting the maxilla [14].

**Figure 6:**
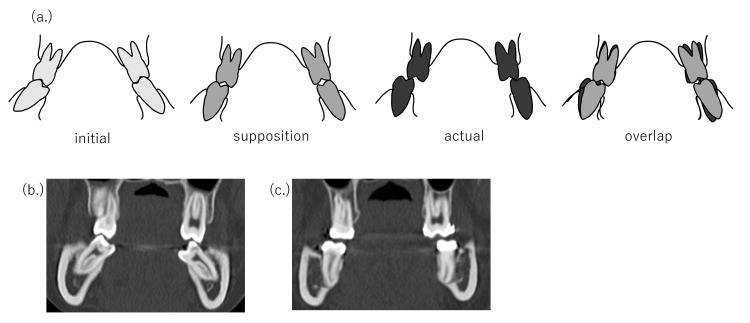
(a) When molars are highly buccolingual compensated, it is common for the dental arch width to change from the expected width when decompensating. As a result, the widths of the upper and lower jaws often do not conform. (b) Initial consultation and (c) just before surgery in a sample case.

**Figure 7:**
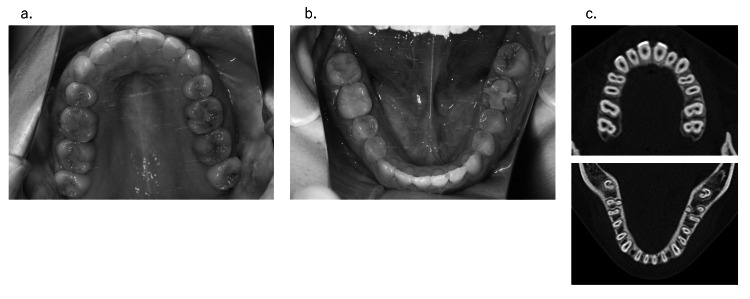
(a, b) Disharmony of the upper and lower arch forms. (c) CT imaging. The upper and lower widths will also not match if the maxillary molar bone is narrow or if the mandible is buccally expanded toward the posterior molar.

**Figure 8:**
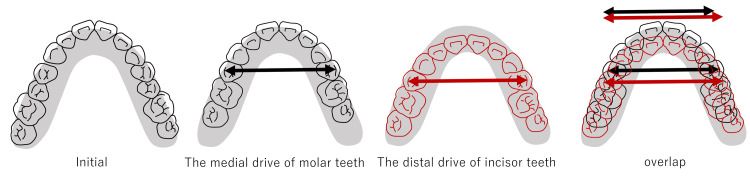
The amount of retraction of the anterior teeth changes the width of the alveolar bone where the molars are located, which also changes the dental arch width.

In 26 of 43 cases (60.4%), the cases were more complicated than the initial plan. However, 13 of 26 patients (50.0%) underwent surgical methods proposed at the initial examination. The factors relating to width issues are listed as follows (in some cases, several factors may be involved). (1) Change in the posterior tooth axis: Preoperative orthodontic treatment sometimes excessively improves the posterior tooth axis compared with the initial plan [15] when molars have severe labial or lingual inclination in either or both the upper and lower jaws (Figure 6). In these cases, it is often necessary to split the maxilla, or conversely, a maxillary split may be no longer necessary. (2) Insufficient expansion by rapid palatal expansion: In relatively young patients, the maxilla may be expanded along the median palatine suture using rapid palatal expansion appliances. In some rare instances, palatal expansion may be difficult. In these cases, surgically assisted rapid palatal expansion (SARPE) or the splitting of the maxilla becomes necessary. (3) Discrepancies between the maxillary and mandibular dental arches: When the maxillary and mandibular dental arch forms are different, discrepancies in the posterior width between the upper and lower jaw are common. Anatomically, the mandibular dental arch widens outward from the posterior mandible. Therefore, if the molars are placed upright in the mandibular alveolus, a horizontal overlap discrepancy may occur if the posterior width of the maxilla is narrower (Figure 7). (4) Differences in the anteroposterior positioning of the incisor teeth from the initial plan: During presurgical orthodontic treatment, the incisor teeth are basically aligned upright in the alveolar bone. Generally, the alveolar bone width is narrow in the anterior and is enlarged toward the posterior (Figure 8). From the above, if the anteroposterior positioning of the incisors is different from the initial plan, the dental arch width in the molar region will change. For example, when the amount of incisal retroclination is less than expected based on the initial plan, the molars are aligned in the anterior position and the dental arch width in the molar region becomes narrower than expected. (5) Insufficiencies relating to the posterior tooth axis and dental arch width with multi-bracket appliances. (6) Preoperative orthodontic treatment often fails to decompensate and achieve appropriate buccal or lingual inclination due to various factors, such as occlusion and habits, even if the initial plan is assumed. In such cases, surgical procedures are used to expand the width of the dental arches and set up a stable occlusion (these cases can be both too wide and too narrow). (7) The molar arch width discrepancy may be compensated for with a prosthesis. For example, surgical expansion by splitting the maxilla may be planned during the initial plan; however, this surgery may not be omitted if adequate horizontal overlap and normal buccal overjet can be established with a prosthesis. (8) The lateral width relationship of the dentition is left untreated, and the lateral crossbite is accepted during the final treatment.

The occlusal condition at the end of the orthodontic treatment is similar to (6). However, the crossbite is left untreated without a prosthesis, and the molar occlusion is established.

Movement Amounts

In patients with surgical changes relating to movement amounts, 18 (94.7%) changed from only mandibular surgery to surgery involving both jaws. In some cases, the excessive amount of mandibular movement required and the possibilities of surgical changes were explained to the patients. In 14 of these cases, presurgical orthodontic treatment was performed as planned, but surgery changed to involve both the maxilla and mandible to stabilize the occlusion. In the remaining four cases, potential surgical changes were not explained to the patient. However, the surgical method was changed because of the movement amount required. In these cases, the lingual movement of both incisors was significantly different from the initial plan.

Differences in the Upper Occlusal Plane Between the Anterior and Posterior Teeth

**Figure 9:**
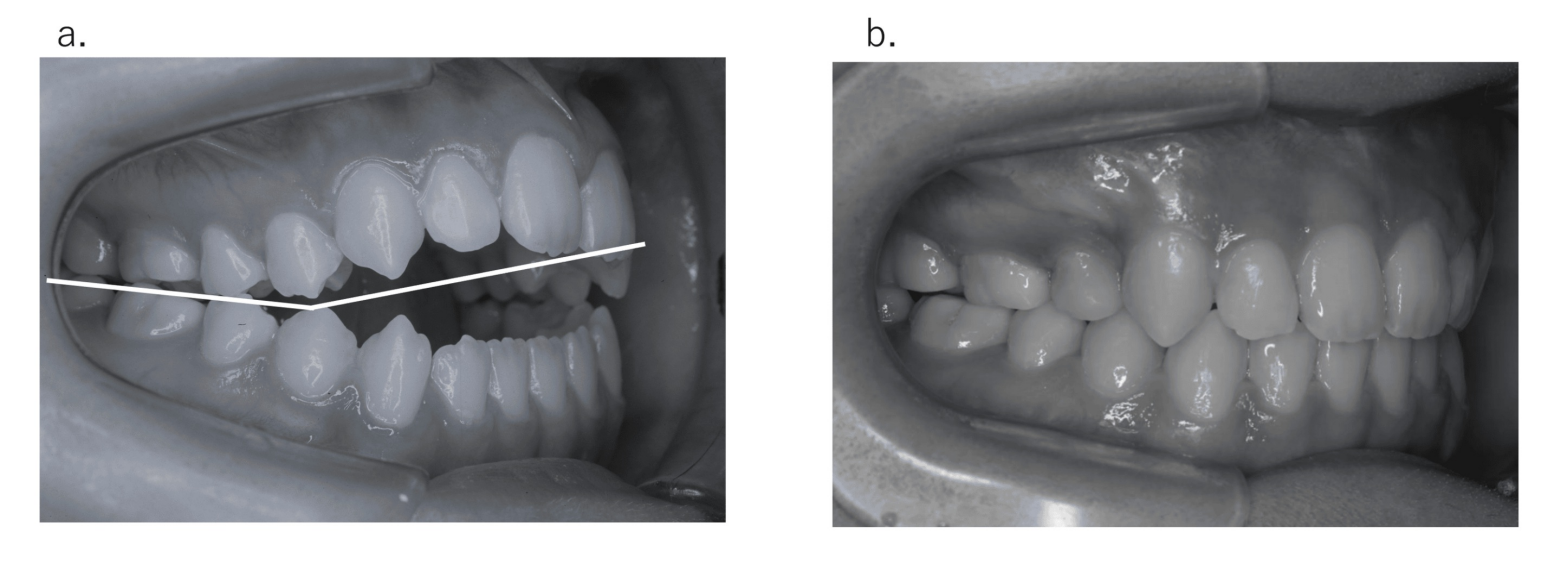
Difference of upper occlusal plane between anterior and posterior teeth. (a) Initial. (b) Debonding. If the open bite is significant, the maxillary anterior teeth may have been compressed for a long period and the alveolar bone may be deformed. If the amount of anterior tooth eruption or molar pressure reduction is significant, a plan may be developed to flatten the occlusal plane surgically.

In patients with maxillary occlusal planes that differ between the incisor and molar regions and the angle between two planes is excessive, the maxillary occlusal plane is often corrected by surgery (Figure 9). If corrected by orthodontic treatment alone, severe incisal elongation and premolar intrusion are required. The maxilla is divided by the anterior and posterior teeth due to the flattened occlusal plane for preventing gingival recession and excessive extension of treatment. However, such an occlusal plane is occasionally improved by tooth extraction and dental arch expansion in presurgical orthodontic treatment.

In this study, 10 cases demonstrated a difference between the incisor and molar regions of the occlusion plane. One had an edge-to-edge occlusion, and the others had an open bite. In all cases, the surgery method performed was not examined.

Extent of Mandibular Rotation in the Sagittal Plane

**Figure 10:**
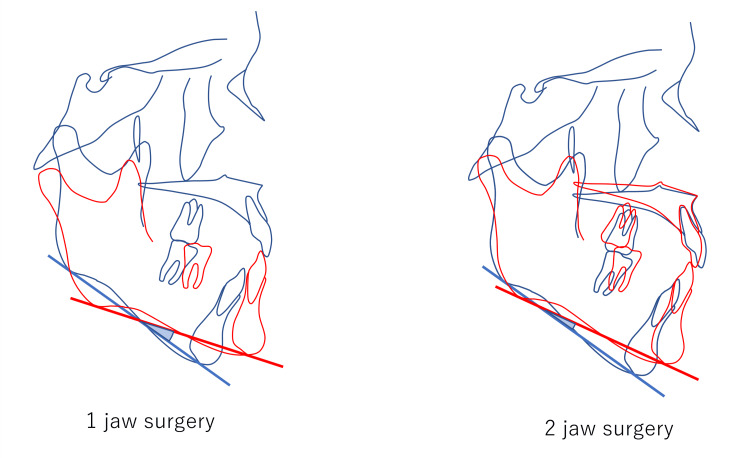
By depressing the maxilla, the counterclockwise rotation of the mandible can be reduced. This can decrease relapse. Blue: before surgery. Red: after surgery.

In 13 cases, the surgical method was changed due to the extent of mandibular rotation in the sagittal plane (Figure 10). Three cases were changed from mandibular surgery only to bimaxillary surgery. The other two cases were not evaluated for potential changes in surgical methods during the initial examination. In our hospital, the basic procedure is bimaxillary surgery when the mandibular plane shows more than 4° of counterclockwise rotation, to prevent retroversion.

Occlusal Cant (the Frontal Occlusal Plane)

Seven cases had surgical changes due to cant inclination. Four cases were evaluated for potential changes in surgical methods during the initial examination, and three cases were not examined.

In one case, the maxilla was not operated on because the patient did not wish to modify the canthotomy. The remaining six patients had an occlusal cant of less than 4° at the time of initial examination (4° is the standard for correcting canting at Hokkaido University and is the threshold where 90% of observers recognize occlusal canting). Due to excessive mandibular deviations, improvement of facial asymmetry was strongly desired; thus, maxillary surgery was added to correct the canting inclination.

Avoidance of Risk Due To the Patient's Condition

Invasive surgery may be contraindicated due to patient conditions, such as systemic disease and pregnancy. Conversely, if airway stricture after mandibular setback surgery is a concern, bimaxillary surgery may be preferred even if it is possible to operate on the mandible only from the perspective of the amount of jaw movement required.

Procedures added or canceled in complicated and simplified cases

**Figure 11:**
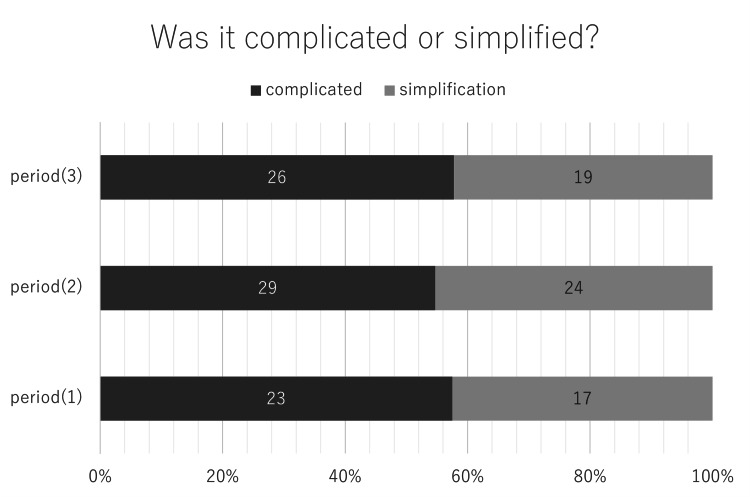
The percentage of orthodontic surgeries that have been complicated or simplified. The complicated percentage or simplification from Periods 1 to 3 remained the same.

There was not a remarkable change in the percentage of complicated and simplified cases per period (Figure 11). Procedures added to complicated cases were LeFort I (38 cases), LeFort I multisegmented (28 cases), segmental osteotomy (10 cases), and corticotomy and distraction (1 case). Among the simplified cases, LeFort I multisegmented was omitted in 27 cases, LeFort I in 23 cases, segmental osteotomy in 9 cases, and SSRO in 2 cases (Table 2)

**Table 2:** Procedures added or canceled in complicated and simplified cases. SSRO: sagittal split ramus osteotomy.

		Period 1	Period 2	Period 3
Complicated (added)	Le Fort I	7	18	14
	Le Fort I multisegmented	13	8	7
	Segmental osteotomy	4	2	4
	Corticotomy	0	0	1
	Destruction	0	1	0
	SSRO	0	0	0
Simple (canceled)	Le Fort I	6	6	11
	Le Fort I multisegmented	11	13	3
	Segmental osteotomy	0	4	5
	Corticotomy	0	0	0
	Destruction	0	0	0
	SSRO	1	1	0

## Discussion

In surgical orthodontic treatment, the orthognathic surgery planned in the initial examination is often changed at the time of surgery. We identified various factors in this study. The most common reason was dental arch width, followed by jaw movement amount required during surgery, the mandibular rotation amount in the sagittal plane, differences in the upper occlusal plane between the anterior and posterior teeth, patient's condition, and cant-related issues. Each factor was, of course, investigated in more detail, and some factors were attributed to presurgical orthodontic treatment, as shown in previous studies [16]. However, the fact that there were many changes related to width reveals a problem not only in the anterior-posterior but also in the transverse of the patient, which has been impeding the presence of upper and lower jaw disharmony. In addition, the commitment to molar occlusion and tooth axis in the surgical orthodontic treatment at Hokkaido University Hospital may have influenced this result.

The importance of preliminary considerations based on occlusal changes due to presurgical orthodontic treatment, evaluation of the canine and molar tooth axis [17], the dental arch form [18], and the alveolar condition at the initial examination [19,20] are indicated in treatment planning [1]. One possible reason for the variation in the percentage of surgical changes over time is the expanding age range among patients [13]. As the average age of patients changes from early 20s to late 20s, there is an increase in the number of missing teeth and prostheses in patients with jaw deformities, as well as an increase in prevalence and aesthetic demands. These factors complicate preoperative treatment and can create surgical uncertainties. Additionally, established treatment criteria regarding the amount of movement, rotation, and canting govern the treatment provided at Hokkaido University Hospital. For example, according to our criteria, if the amount of required movement exceeds 10 mm, the rotation of the mandible exceeds 4°, or if the cant exceeds 4°, then the upper and lower jaws should be operated on. The initial plans often aimed to enforce these standards. Moreover, we do not perform tri-segmental maxillary surgery as often anymore. Instead, we are now actively performing segmental osteotomies. Notably, the biggest factor influencing differences between the initial treatment plan and the surgery performed is the introduction of the orthodontic anchoring screw to health insurance in April 2014 in Japan (during Period 2). Anchoring screws made it possible to move anterior teeth and depress molars, which had been difficult before [21,22]. As a result, treatment plan changes increased. 

Regarding the changes in the surgical method, the cases that require attention are limited. Cases involving decreased invasiveness of the surgical methods are not often problematic. For example, the planned bimaxillary surgical changes to mandibular surgery only, or the planned maxillary split becomes unnecessary, and so on. Such cases are welcomed by patients and surgeons. Cases that are planned as mandibular surgery only during the initial examination but subsequently changed to bimaxillary surgery are also not a problem if the possibility of bimaxillary surgery is explained to the patient during the initial examination.

The cases that become problematic are usually those involving more complex and more invasive surgery, but only simple and easy surgical methods were planned and explained to the patient during the initial examination. In such cases, sufficient explanation regarding the need for more complex surgery and the associated risks, as well as sufficient psychological care, are necessary, especially for patients who may be nervous before orthognathic surgery [23-25]. We believe that re-evaluating the status of preoperative orthodontic treatment and considering revisions to achieve improved functional and aesthetic outcomes are thought to enhance patient satisfaction. However, it is not possible to objectively assess how much the postoperative occlusion improved, shortened treatment time, or to what extent patient satisfaction increased as a result of these modifications. This is because a direct comparison with outcomes based on the original surgical plan is not feasible. This limitation should be recognized as a constraint of the present study.

In surgical orthodontic treatment, instead of strictly adhering to the initial plan, the treatment plan should be reviewed just before orthognathic surgery, especially the dental arch width and the amount of jaw movement required during surgery. By conducting a preoperative review of the treatment plan, the burden on patients is reduced and the stability of the occlusion is optimized [26-28]. Clinicians must continually reconsider how to provide safer and more effective care to patients.

## Conclusions

This study investigated the changes between the initial treatment plans for jaw deformities and the plans reviewed just before surgery following preoperative orthodontic treatment. It was found that 20%-30% of patients ultimately underwent a surgical procedure different from what was originally planned. These changes are influenced by the widening age range of patients and ongoing advancements in surgical techniques and preoperative correction methods. Key considerations in surgical planning include the width of the jaw, the amount of movement, and the degree of mandibular rotation. Since these factors may change during preoperative orthodontic treatment, it is essential to inform the patients in advance about the potential for modifications to the surgical plan.

The initial treatment plan should not be followed rigidly; instead, surgical planning should prioritize minimizing the patient’s burden and achieving stable occlusion.

## References

[1] Kim YK (2017). Complications associated with orthognathic surgery. J Korean Assoc Oral Maxillofac Surg.

[2] Vig KD, Ellis E III (1990). Diagnosis and treatment planning for the surgical-orthodontic patient. Dent Clin North Am.

[3] Burden D, Mullally B, Sandler J (2001). Orthodontic treatment of patients with medical disorders. Eur J Orthod.

[4] Mathews DP, Kokich VG (1997). Managing treatment for the orthodontic patient with periodontal problems. Semin Orthod.

[5] Shamnur N, Kumar A, Kumar A, Phadkule S (2014). The effect of surgical mandibular advancement on the pharyngeal airway dimensions: a cephalometric study. J Ind Orthod Society.

[6] Proffit WR, Phillips C, Turvey TA (2012). Stability after mandibular setback: mandible-only versus 2-jaw surgery. J Oral Maxillofac Surg.

[7] Choi SH, Jeon JY, Lee KJ, Hwang CJ (2021). Clinical applications of miniscrews that broaden the scope of non-surgical orthodontic treatment. Orthod Craniofac Res.

[8] Matsushita K, Yamaguchi HO, Koshikawa-Matsuno M, Inoue N (2014). Evaluation of a three-stage method for improving mandibular retrognathia with labially inclined incisors using genioplasty, segmental osteotomy, and two-jaw surgery. Case Rep Med.

[9] Nagasaka-Konno M, Iijima M, Horii G, Shibata T, Mizoguchi I (2016). Mandibular symphyseal distraction followed by class III surgical orthodontic treatment: a case report. J World Fed Orthod.

[10] Baik UB, Han KH, Yoo SJ, Park JU, Kook YA (2013). Combined multisegmental surgical-orthodontic treatment of bialveolar protrusion and chin retrusion with severe facial asymmetry. Am J Orthod Dentofacial Orthop.

[11] Proffit WR, White RP Jr (2015). Combined surgical-orthodontic treatment: how did it evolve and what are the best practices now?. Am J Orthod Dentofacial Orthop.

[12] Katagiri W, Kobayashi M, Sasaki A (2020). Survey on the current state of surgical orthodontic treatment in Japan-results of the 2017 survey by the Japanese Society for Jaw Deformities (Article in Japanese). Jpn J Jaw Deform.

[13] Hasebe D, Suda D, Asai Y, Kojima T, Kato Y, Kobayashi M (2016). Clinical analysis of orthognathic surgeries performed over the past 48 years in the Department of Reconstructive Oral Surgery, Niigata University Graduate School of Medical and Dental Sciences (Article in Japanese). Jpn J Jaw Deform.

[14] Southard TE, Marshall SD, Allareddy V, Shin K (2019). Adult transverse diagnosis and treatment: a case-based review. Semin Orthod.

[15] Nakamura Y, Miyamoto Y, Kanzaki H, Wada S (2015). Orthodontic treatment of an adult class III malocclusion with severe transverse dental compensation by remaining of buccal crossbite. Int J Orthod Milwaukee.

[16] Jheon AH, Oberoi S, Solem RC, Kapila S (2017). Moving towards precision orthodontics: an evolving paradigm shift in the planning and delivery of customized orthodontic therapy. Orthod Craniofac Res.

[17] Tanne K, Nagataki T, Inoue Y, Sakuda M, Burstone CJ (1991). Patterns of initial tooth displacements associated with various root lengths and alveolar bone heights. Am J Orthod Dentofacial Orthop.

[18] Lee SJ, Kim TW, Nahm DS (2006). Transverse implications of maxillary premolar extraction in Class III presurgical orthodontic treatment. Am J Orthod Dentofacial Orthop.

[19] Sun B, Tang J, Xiao P, Ding Y (2015). Presurgical orthodontic decompensation alters alveolar bone condition around mandibular incisors in adults with skeletal Class III malocclusion. Int J Clin Exp Med.

[20] Chung CJ, Jung S, Baik HS (2008). Morphological characteristics of the symphyseal region in adult skeletal Class III crossbite and openbite malocclusions. Angle Orthod.

[21] Heymann GC, Tulloch JF (2006). Implantable devices as orthodontic anchorage: a review of current treatment modalities. J Esthet Restor Dent.

[22] Mohamed RN, Basha S, Al-Thomali Y (2018). Maxillary molar distalization with miniscrew-supported appliances in class II malocclusion: a systematic review. Angle Orthod.

[23] Thastum M, Andersen K, Rude K, Nørholt SE, Blomlöf J (2016). Factors influencing intraoperative blood loss in orthognathic surgery. Int J Oral Maxillofac Surg.

[24] Espínola LV, D'ávila RP, Landes CA, Ferraz EP, Luz JG (2022). Do the stages of orthodontic-surgical treatment affect patients' quality of life and self-esteem?. J Stomatol Oral Maxillofac Surg.

[25] Takatsuji H, Kobayashi T, Kojima T, Hasebe D, Izumi N, Saito I, Saito C (2015). Effects of orthognathic surgery on psychological status of patients with jaw deformities. Int J Oral Maxillofac Surg.

[26] Teittinen M, Tuovinen V, Tammela L, Schätzle M, Peltomäki T (2012). Long-term stability of anterior open bite closure corrected by surgical-orthodontic treatment. Eur J Orthod.

[27] de Lir AD, de Moura WL, Ruellas AC, Souza MM, Nojima LI (2013). Long-term skeletal and profile stability after surgical-orthodontic treatment of class II and class III malocclusion. J Craniomaxillofac Surg.

[28] Koerich L, Ruellas AC, Paniagua B, Styner M, Turvey T, Cevidanes LH (2016). Three-dimensional regional displacement after surgical-orthodontic correction of class III malocclusion. Orthod Craniofac Res.

